# Identification of candidate predictive and surrogate molecular markers for dasatinib in prostate cancer: rationale for patient selection and efficacy monitoring

**DOI:** 10.1186/gb-2007-8-11-r255

**Published:** 2007-11-29

**Authors:** Xi-De Wang, Karen Reeves, Feng R Luo, Li-An Xu, Francis Lee, Edwin Clark, Fei Huang

**Affiliations:** 1 Pharmaceutical Research Institute, Bristol-Myers Squibb, Princeton, New Jersey, 08543, USA

## Abstract

**Background:**

Dasatinib is a potent, multi-targeted kinase inhibitor that was recently approved for treatment of chronic myelogenous leukemia resistant to imatinib. To aid the clinical development of dasatinib in prostate cancer, we utilized preclinical models to identify potential molecular markers for patient stratification and efficacy monitoring.

**Results:**

Using gene expression profiling, we first identified 174 genes whose expression was highly correlated with *in vitro *sensitivity of 16 cell lines and, thus, considered as candidate efficacy predictive markers. Among these are important prostatic cell lineage markers, cytokeratin 5, androgen receptor and prostate specific antigen. Our results indicate that 'basal type' cell lines with high expression of cytokeratin 5 and low expression of androgen receptor or prostate specific antigen are sensitive to dasatinib. To identify markers as surrogates for biological activity, we treated cell lines with dasatinib and identified genes whose expression was significantly modulated by the drug. Ten genes, including that encoding urokinase-type plasminogen activator (*uPA*), were found to not only be potential efficacy markers but also to have reduced expression upon dasatinib treatment. The down-regulation of uPA by dasatinib was drug-specific and correlated with the sensitivity of cell lines to dasatinib. Furthermore, EphA2, a target of dasatinib, was found to be a sensitivity biomarker.

**Conclusion:**

Using the gene expression profiling approach and preclinical models, we have identified prostatic biomarkers that are associated with sensitivity to dasatinib. This study has provided a basis for clinical evaluation of a potential dasatinib efficacy signature in prostate cancer.

## Abstract 2

Gene expression profiling was used to identify genes associated with sensitivity to the tyrosine kinase drug Dasatinib in prostate cancer cell lines, revealing a possible Dasatinib efficacy signature in prostate cancer.

## Background

Prostate cancer is the most common type of cancer in men of western countries. It is estimated that each year about 230,000 men in the United States alone are diagnosed with prostate cancer and approximately 30,000 die of this disease [1]. Although targeted therapeutics have shown promise for cancer patients, their use in treating prostate cancer is still limited. Current regimens available for prostate cancer patients include conventional surgery, radiation and hormonal therapies for early stage tumors, and taxane-based chemotherapy for late stage metastatic tumors [2,3]. There is a clear unmet medical need to develop targeted therapeutics for prostate cancer.

Biomarkers can dictate the successful clinical development of novel anti-cancer drugs and the clinical benefits that patients can derive from -targeted therapeutics. Using expression of HER2 as a patient selection criterion has allowed the successful development of trastuzumab, a monoclonal antibody therapy targeting HER2 in breast cancer. Breast cancer patients identified to over-express HER2 who subsequently receive this therapy show a significant response rate and profound clinical benefits [4]. In contrast, failure to identify and use robust biomarkers in trials for innovative medicines can result in failed approval and/or dramatically delayed clinical development [5,6]. Such examples highlight the need in clinical development to identify molecular biomarkers that will guide patient selection and enable monitoring of drug efficacy at the molecular level.

Dasatinib is a potent, orally available small molecule inhibitor that targets multiple cytosolic or membrane-bound tyrosine kinases, including Src-family kinases (SFKs), Bcr-Abl, c-kit, platelet-derived growth factor receptor (PDGFR) β and EphA2 [7,8]. Due to its potency against leukemic cancer cell lines harboring *BCR-ABL *mutations [9], the clear and imminent need for overcoming imatinib resistance, and the profound clinical benefit demonstrated in clinical trials, dasatinib was recently approved for use in chronic myelogenous leukemia and Philadelphia chromosome-positive acute lymphoblastic leukemia that are resistant or intolerant to imatinib [10]. In contrast, other targets of dasatinib (for example, SFKs, EphA2) have yet to be clinically validated. The involvement of SFKs in a number of cellular processes, such as cell migration, adhesion and angiogenesis, as well as participation of SFKs in a number of clinically relevant pathways (for example, the epidermal growth factor receptor (EGFR) pathway) [11,12] have prompted investigations into the potential use of dasatinib in solid tumors [13]. Such investigations would, as discussed above, best be supported by the use of molecular biomarkers.

To support the development of dasatinib for use in prostate cancer we employed prostate cancer cell lines as preclinical models to identify molecular biomarkers whose expression correlated with the sensitivity to dasatinib and could potentially be used as surrogates to monitor the biological effects of dasatinib in patients. First, we identified candidate predictor genes with baseline expression levels correlated with sensitivity to dasatinib. Next we identified genes that were significantly modulated by dasatinib treatment. Urokinase-type plasminogen activator (*uPA*) was observed to be on both lists, suggesting it may be a candidate predictive and 'surrogate' biomarker. Additionally, EphA2, a target of dasatinib, is a candidate predictor of efficacy in both prostate and breast cancer. Finally, the observed sensitivity to dasatinib of prostate cancer cell lines with expression of basal cell markers, together with a similar observation in breast cancer cell lines [8], suggests a common mechanism of sensitivity to SFKs and a role of SFKs in epithelial tumors derived from the basal layer.

## Results

### Identification of markers correlated with dasatinib sensitivity

**Figure 1:**
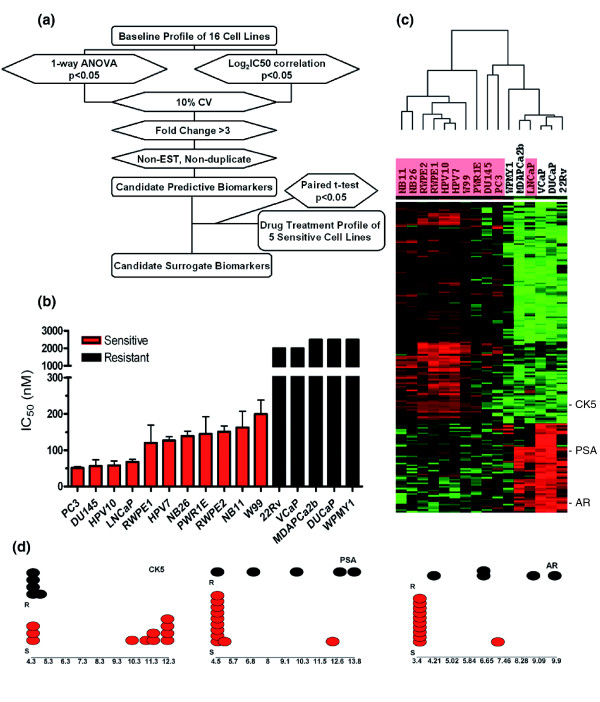
Identification of biomarkers correlated with sensitivity to dasatinib. **(a) **Discovery strategy to identify potential predictive and surrogate biomarkers. **(b) **IC_50 _determination and sensitivity classification of 16 prostate cancer cell lines to dasatinib. **(c) **Cluster analysis showing the relative expression pattern of 174 genes that were highly correlated with dasatinib sensitivity/resistance classification of 16 cell lines. The dasatinib-sensitive cell lines are highlighted in red, and the position of three important prostatic cell markers, *CK5*, *PSA *and *AR*, are marked on the heatmap. These genes have differential expression of more than three-fold between the sensitive and resistant groups. **(d) **Relative baseline gene expression of *CK5*, *PSA *and *AR *in the 16 cell lines. The resistant cells are in black and the sensitive cells are in red. The values on the x-axes are expression level in log_2_-scale.

The aim of this study was to identify both predictive and surrogate biomarkers that could potentially assist the clinical development of dasatinib in prostate cancer. As outlined in Figure 1a, our strategy was first to identify genes whose baseline expression levels correlated with drug sensitivity and passed additional variation requirements to obtain a candidate predictive marker list. Then genes whose expression was modulated by dasatinib in a drug treatment study were identified and compared to the candidate predictive biomarker list to identify genes that were not only correlated with drug sensitivity but also modulated by drug treatment.

The half maximal inhibitory concentration (IC_50_) values of 16 prostate cancer cell lines to dasatinib were determined as shown in Figure 1b. Based on the IC_50 _values, cell lines were classified into two groups: 11 cell lines with IC_50 _values lower than 200 nM (within the range of dasatinib plasma concentrations clinically achieved) were designated as sensitive; and 5 cell lines with IC_50 _values greater than or equal to 2 μM (greater than the highest drug concentration clinically achieved in plasma) were considered resistant. It is noted that the sensitivity or resistance of cell lines is not correlated with their doubling times, as both the sensitive and resistant groups consist of cell lines that grow fast or relatively slowly.

Baseline gene expression profiling of the 16 cell lines was performed using Affymetrix gene chips. Two statistical tests (one-way ANOVA and correlation to log_2_IC_50 _values) were performed to identify genes that were differentially expressed between sensitive and resistant groups (5,961 probe sets with *p *< 0.05) and those that were highly correlated with IC_50 _(4,575 probe sets with *p *< 0.05), respectively; 4,248 probe sets overlapped in these two analyses, suggesting that the categorization of sensitive and resistant groups reflected well the sensitivity of the cells (IC_50_) to dasatinib. The list was further filtered with a requirement for a 10% coefficient of variation (CV) across all samples and a minimum 3-fold differential expression between the sensitive and resistant groups, resulting in the selection of 213 probe sets. Expressed sequence tags (ESTs) and duplicate probe sets were further removed to generate the candidate predictive marker list of 174 genes (Additional data file 1).

The expression pattern of these 174 genes on the 16 cell lines was visualized by cluster analysis. As shown in Figure 1c, these 16 cell lines were separated into two major groups (the dasatinib-sensitive cell lines are marked in red). Interestingly, the cell lines in the left cluster were all sensitive to dasatinib; within the right cluster, DU145, PC3 and LNCaP cells were also highly sensitive to dasatinib.

Genes of biological interest in Additional data file 1 include EGFR pathway genes such as amphiregulin and epiregulin, transforming growth factor pathway genes such as *TGFα*, *TGFβ2 *and *TGFβRII*, as well as other receptor tyrosine kinases, such as the *Met *proto-oncogene and fibroblast growth factor receptor 2. These genes were more highly expressed in sensitive cell lines. Most strikingly, several important prostatic cell markers, such prostate specific antigen (PSA; also known as kallikrein 3) and androgen receptor (AR) were over-expressed in the resistant cell lines, while cytokeratin (CK) 5 was highly expressed in the sensitive cell lines (Figure 1c).

The relative expression levels of *CK5*, *PSA *and *AR *in these 16 cell lines are shown in more detail in Figure 1d. We observed that resistant cell lines all express very low levels of *CK5 *and sensitive cell lines all express high levels of *CK5*, except for DU145, PC3 and LNCaP cells (Figure 1c). As CK5 is a basal cell marker for the prostatic cell lineage, these data suggest that cells exhibiting the basal phenotype are sensitive to dasatinib and that cells expressing lower levels of CK5 tend to be resistant. The expression pattern of PSA and AR, two luminal cell markers, complementarily reinforces the above observation. While higher expression of *PSA *and *AR *is correlated with drug resistance, lower expression of these two genes is correlated with dasatinib sensitivity. The LNCaP cell line, which was sensitive to dasatinib and expressed high levels of *PSA *and *AR*, is the only exception to this observation out of five cell lines (MDAPCa2b, LNCaP, VCaP, DUCaP, and 22Rv) found to express higher levels of *PSA *and *AR *(Figure 1c).

### Identification of markers that are also modulated by dasatinib treatment

Five dasatinib-sensitive cell lines, including two *CK5*-expressing (PWR1E and RWPE2) and three *CK5*-nonexpressing cell lines (PC3, DU145 and LNCaP), were treated with dasatinib. Comparison by paired *t*-test of gene expression profiles between post-dasatinib treatment and mock treatment for each cell line revealed that 1,628 probe sets were significantly modulated by drug treatment (*p *< 0.05). Comparison of these 1,628 probe sets with the list of candidate predictive markers (Additional data file 1) indicated that 10 genes were common to both lists. These ten genes, which may be potentially used to predict sensitivity to dasatinib and to serve as surrogates for drug activity, are indicated in the last column of Additional data file 1. Interestingly, all ten of these genes were highly expressed in sensitive cell lines and decreased in expression after dasatinib treatment. These genes include those encoding epiregulin, a component in the EGFR pathway, FHL2 and AXL kinases, and uPA. Three of these ten genes including *LAMC2*, *EREG *and *uPA *encode proteins that are secreted to the extracellular matrix.. This set of genes may represent genes whose expression are under the regulation of genes targeted by dasatinib.

### Common biomarkers identified in prostate and breast preclinical studies

To facilitate clinical development of dasatinib for breast cancer, a similar preclinical biomarker study was performed in this laboratory [8]. Biomarkers predictive of dasatinib sensitivity in breast cancer cell lines were identified and are currently being assessed in clinical trials. Since the majority of breast tumors are also epithelial in origin, we compared the biomarkers discovered in the current prostate cancer study with those identified in the breast cancer study. To this end, in addition to the 174 genes noted above, we also included probe sets in the list of 1,475 probe sets (after the 10% CV step, but before the fold-change >3 filter step) that were also significantly modulated by dasatinib (that is, present in the list of 1,628 probes) as candidate prostate biomarkers and compared them to the breast cancer biomarker list of 161 genes [8]. Fourteen genes were identified as common biomarkers in both tissue types (Additional data file 2). Notably, *EphA2*, a dasatinib target, was significantly correlated with dasatinib sensitivity in both prostate and breast cancer cell lines. As shown in Additional data file 2, the mean expression of *EphA2 *was significantly higher in sensitive cell lines than in resistant cell lines (2.69-fold with *p *= 0.005 by *t*-test).

**Figure 2:**
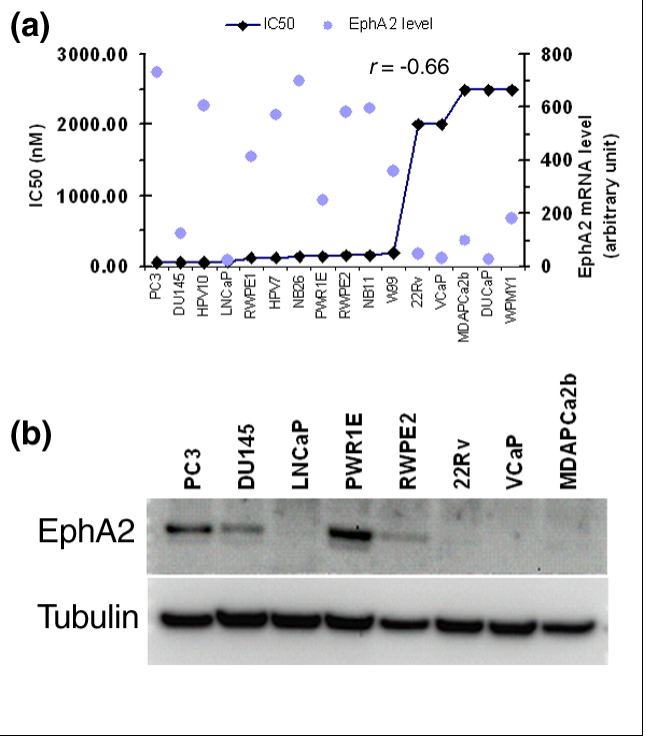
Correlation of *EphA2 *gene expression with sensitivity to dasatinib. **(a) **Negative correlation between the expression levels of *EphA2 *(black diamonds) and the IC_50 _(gray circles) values for the 16 cell lines. The coefficient of the Pearson correlation is -0.66, indicating a high reverse correlation. **(b) **Expression of EphA2 protein in five sensitive and three resistant cell lines. Overall, the protein expression levels in these cell lines correlated well with the mRNA levels detected by microarray analysis.

As shown in Figure 2a, the expression level of *EphA2*, as detected by microarray baseline profiling, was correlated with the IC_50 _values of the prostate cell lines (higher *EphA2 *expression with lower IC_50 _or high sensitivity, Pearson correlation coefficient = -0.66). As a validation, we also performed western blot analysis to examine the expression of EphA2 protein in five sensitive and three resistant cell lines (Figure 2b). Overall, those cell lines with higher levels of *EphA2 *mRNA expressed relatively higher levels of EphA2 protein, indicating good concordance between gene and protein expression for EphA2. While the *EphA2 *RNA level in DU145 cells was relatively low compared to the other sensitive cell lines, its protein level appeared comparable, suggesting that *EphA2 *expression is also regulated at the protein translation or stabilization levels. Our western blot results on EphA2 protein in PC3, DU145 and LNCaP cells are consistent with a previous report [14]. With the correlation of its expression with dasatinib sensitivity in cell lines, and being a target of the drug, EphA2 appears to be a strong candidate biomarker for dasatinib in prostate cancer.

### Down-regulation of uPA expression by dasatinib

**Figure 3:**
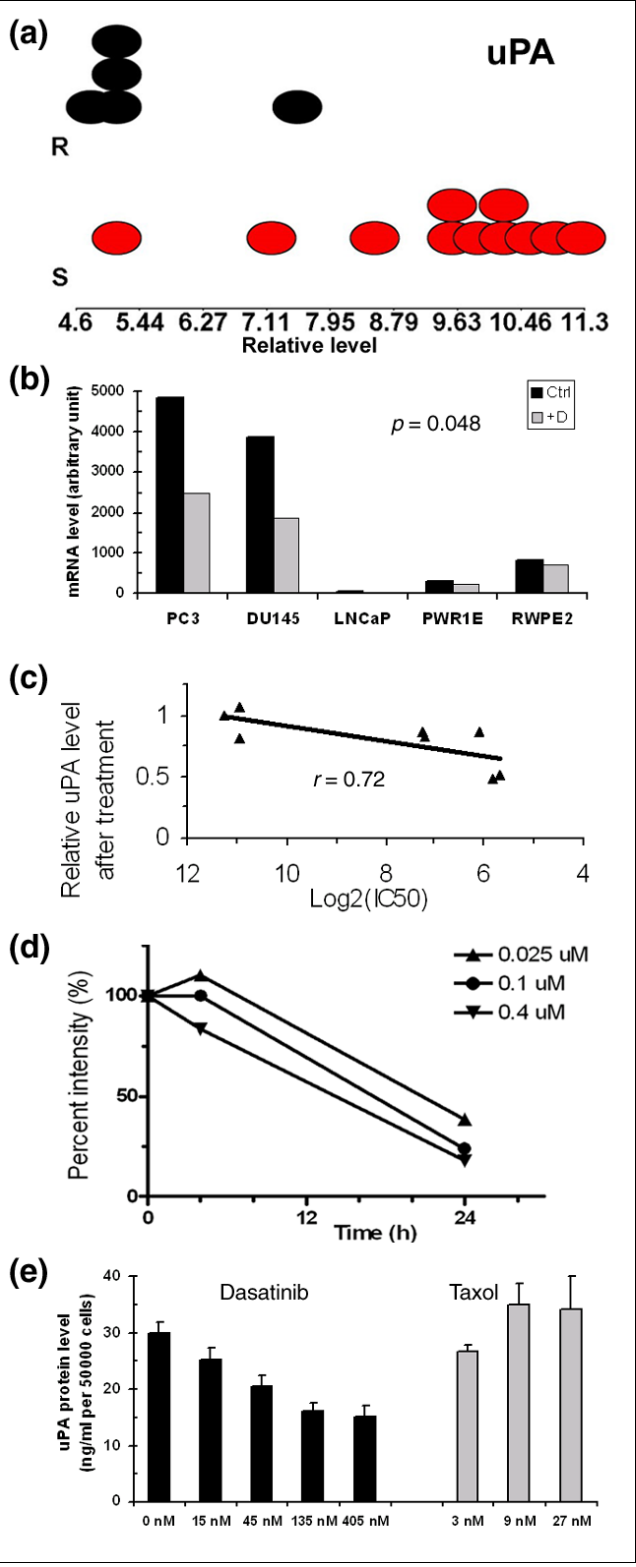
*uPA *gene expression and regulation by dasatinib analyzed at mRNA and protein levels. **(a) **Differential baseline expression of *uPA *gene between resistant (R, in black) and sensitive (S, in red) cell lines. The x-axis values are the expression level in log_2_-scale. The resistant cell line expressing a high level of *uPA *was WPMY1 and the sensitive cell line expressing a low level of *uPA *was LNCaP. **(b) **Down-regulation of *uPA *mRNA level by dasatinib treatment in five sensitive cell lines. The cells were treated with 100 nM dasatinib (+D) or DMSO (Ctrl) for 48 h. The *p *value was 0.048 by paired *t*-test, indicating a significant reduction of *uPA *mRNA following dasatinib treatment. **(c) **Correlation between dasatinib-induced *uPA *mRNA down-regulation with the sensitivity of cell lines to dasatinib. In addition to the five sensitive cell lines, three dasatinib-resistant cell lines, 22Rv, MDAPCa2b, and VCaP, were also treated with dasatinib as in (b). The extent of *uPA *down-regulation by dasatinib (y-axis) was negatively correlated with the log_2_IC_50 _values (x-axis) of these eight cell lines. **(d) **Dose-dependent down-regulation of *uPA *mRNA expression in PC3 cells. Cells were treated with or without different concentrations of dasatinib for 4 or 24 h. The *uPA *expression level relative to control is shown on the y-axis. **(e) **Dose-dependent inhibition of secreted uPA protein in PC3 cells by dasatinib but not by paclitaxel. Cells were treated with different doses of dasatinib, paclitaxel or DMSO for 24 h. The amount of uPA protein secreted into the culture medium by 50,000 viable cells was assessed by ELISA assay.

Since the secreted protein uPA is regulated by SFKs [15], we further evaluated the expression of the *uPA *gene and its modulation by dasatinib. As shown in Figure 3a (and also Additional data file 1), the expression level of the *uPA *gene in sensitive cell lines was significantly higher than in resistant cell lines. A second probe set for the *uPA *gene also showed a similar expression pattern. Additionally, three probe sets for the uPA receptor, which partners with uPA in its function, all showed a similar expression pattern as *uPA *in these cell lines (data not shown).

The down-regulation of *uPA *mRNA expression upon dasatinib treatment was observed (Figure 3b). A relatively mild reduction of *uPA *expression was observed in two CK5-expressing cells (PWR1E and RWPE2) while the reduction in PC3 and DU145 cells was much stronger (approximately 50%). LNCaP cells, which express a much lower level of *uPA*, also showed a reduction upon dasatinib treatment. When we extended the same drug treatment study in three resistant cell lines, 22Rv, VCaP and MDAPCa2b (Figure 3c), the magnitude of *uPA *reduction by dasatinib was correlated nicely with the sensitivity of cells to dasatinib (*r *= 0.72), with the highest reduction seen in the most sensitive cell line. This suggests uPA is a potential surrogate biomarker for the biological effect of dasatinib. Furthermore, in a multiple-dose treatment study with PC3 cells, we found that the reduction of *uPA *mRNA level by dasatinib at 4 h was minimal for all doses compared to untreated control, but the changes were dramatic at 24 h and occurred in a dose-dependent fashion (Figure 3d).

The down-regulation of uPA expression by dasatinib was also seen at the protein level. Using an enzyme-linked immunosorbent assay (ELISA), we found in a time course experiment that the amount of uPA protein secreted by PC3 cells into the growth medium after 24 h was reduced by dasatinib treatment, and the extent of this reduction was dose-dependent, as shown in Figure 3e. As a control, when using a cytotoxic agent, paclitaxel, we did not see a dose-dependent reduction in the secreted uPA protein level, suggesting that down-regulation of uPA expression is not a consequence of cell growth inhibition.

### Rationale for patient stratification in dasatinib prostate cancer trials

**Figure 4:**
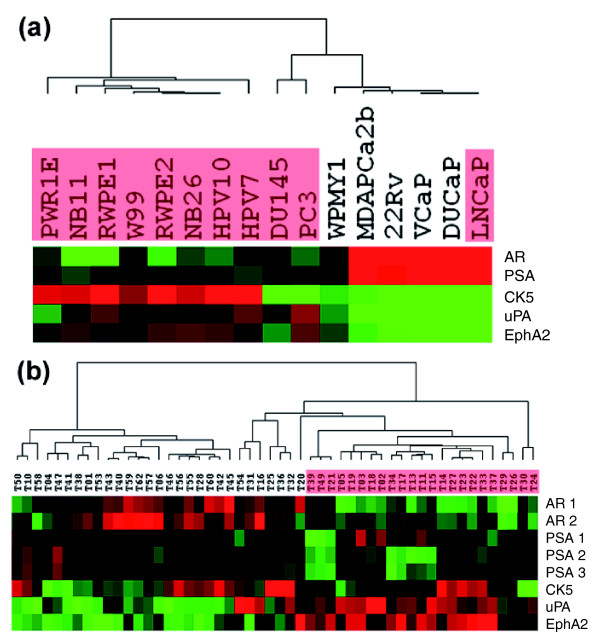
Expression pattern of a five-gene model in prostate cell lines and tumors. **(a) **Hierarchical clustering of five genes, *AR*, *PSA*, *CK5*, *uPA*, and *EphA2*, in prostate cell lines. AR and PSA are two luminal cell markers, and CK5 is a basal cell marker. The resistant cell lines were separated from the majority of the sensitive cells (labeled in red) by these five genes. Note the high expression of *CK5 *and/or *uPA*, and *EphA2 *in sensitive cells and the nearly opposite expression pattern in resistant cells. **(b) **The expression pattern of these 5 genes in 52 prostate tumors. The gene expression of this published data set was profiled on Affymetrix HG-U95 gene chips. Two *AR*, three *PSA *probe sets and one *CK5*, *uPA *and *EphA2 *probe set are available on the chip and were retrieved for cluster analysis. The tumor samples exhibiting a dasatinib-sensitive pattern, that is, with low *AR *and *PSA *expression and high *CK5 *and/or *uPA*, and *EphA2 *expression are highlighted in red.

Based on their differential expression, we reasoned that CK5, PSA, and AR could serve as predictive biomarkers for identification of subtypes of prostate tumors that would benefit from dasatinib treatment. We also reasoned that uPA and EphA2 could potentially be used as markers to monitor dasatinib activity because of their correlation with drug sensitivity and the links with dasatinib's mechanisms of action. The expression patterns of these 5 genes in the 16 cell lines are shown in Figure 4a. Five dasatinib resistant cell lines, WPMY1, MDAPCa2b, 22Rv, VCaP, and DUCaP, all expressed high levels of *AR *and *PSA *and low levels of *CK5*, *uPA *and *EphA2*. In contrast, sensitive cell lines expressed low levels of *AR *and *PSA*, with the exception of LNCaP, and high levels of *uPA*, *EphA2 *and/or *CK5*.

The dynamic range in the expression of these 5 genes and the approximate patient population exhibiting dasatinib-responsive expression patterns were examined using a previously published prostate tumor data set consisting of 52 tumor samples [16]. As shown in Figure 4b, nearly 44% (23/52, sample ID labeled in red) of the prostate tumors showed the 'dasatinib-responsive' expression patterns (that is, low *AR *and *PSA *and high *uPA*, *EphA2 *and/or *CK5*). In the remaining approximately 56% of tumors, the expression of *AR *and *PSA *were concordantly relatively high and the expression of *uPA *and *EphA2 *were relatively low. There were certain degrees of co-expression as well as mutually exclusive expression of *AR *and *CK5 *in this data set, reminiscent of the expression pattern of these two genes in basal, intermediate and luminal cells of normal prostatic epithelium [17,18].

## Discussion

The ideal scenario for identifying biomarkers for clinical use is to use samples obtained from patients undergoing therapy with the investigational drug and to analyze gene expression data in the context of patient response data. Since dasatinib is a novel agent in early clinical development, using preclinical models to identify candidate biomarkers for assisting clinical development appears the best option. In this study, we used 16 prostate cell lines to identify biomarkers that were correlated with the sensitivity of cells and with the mechanisms of drug action. These biomarkers could potentially be used for predicting and monitoring dasatinib response. In particular, we identified five genes (*AR*, *PSA*, *CK5*, *uPA *and *EphA2*) that were highly associated with drug sensitivity/resistance and/or modulated by drug treatment. Consistent with our observation in breast cancer cell lines [8], it appears that basal-type prostate cancer cells expressing low levels of *AR *and *PSA *and a high level of *CK5 *are most responsive to dasatinib treatment. Higher expression levels of *uPA *and *EphA2 *may also help to define patients that will benefit from dasatinib treatment. In addition, *uPA *expression was regulated by dasatinib and such regulation was highly correlated with the sensitivity of cells to dasatinib, suggesting that *uPA *expression can potentially be used as a surrogate marker to monitor dasatinib activity. As a potent inhibitor against Abl, c-kit, PDGFRβ and, in particular, Src and EphA2, which have been shown to play important roles in prostatic tumorigenesis [13,14], dasatinib holds high promise as a potential treatment for prostate cancer. It is noted that imatinib, which inhibits three of these targets, including Abl, c-kit and PDGFR kinases, showed minimal efficacy in early clinical testing with a small number of patients [19,20]. Identification of these preclinical candidate markers and further validation of them in early clinical trials would facilitate patient stratification in registration trials of dasatinib.

In our data analysis, we used an approach that emphasized both statistical significance and high-fold differential gene expression between subgroups. Since we have only 16 cell lines, a pool that may not be necessarily large enough for stringent statistical analyses, we included other requirements, including stringent variation and fold change filters. Notably, the approach we undertook was essentially consistent with that reported by a recent publication from the MicroArray Quality Control initiative led by the Food and Drug Administration, which showed that gene lists ranked by fold change and filtered with non-stringent yet statistically significant tests were more reproducible across platforms than lists generated with other analytical strategies [21]. The result we obtained is biologically meaningful as we identified subtypes of cells sensitive to dasatinib that reflect normal and/or pathogenic prostatic biology and genes that reflect the function of dasatinib targets. In addition, a number of biomarkers identified were also observed from a recently published breast cancer study [8].

Inside normal prostatic epithelium, there exist two major types of epithelial cells, basal and luminal epithelial cells. Several recent studies suggest that there may be further divisions of epithelial cells into subtypes that are more specialized in function. Among these subtypes are prostatic stem cells, transit amplifying cells, intermediate cells and secretary luminal cells, which can be differentiated based on their expression pattern of certain cellular markers. In our study, we found that cells with low *AR *and *PSA *expression and high *CK5 *expression represent a sub-population that is sensitive to dasatinib. This expression pattern matches that of epithelial cells residing in the basal compartment, which can potentially be prostatic stem cells or transit amplifying cells. Since these two types of cells are able to self-renew and quickly proliferate to give rise to new or more differentiated cells, an intrinsic relationship may exist between the expression and function of dasatinib targets such as the SFK LYN and the proliferation of these cells [11]. Indeed, LYN is more highly expressed in the basal layer than in the luminal layer, and in tumors LYN tends to be more highly expressed in less differentiated regions [22]. Our result that basal type prostatic cells are sensitive to dasatinib resonates with recent reports showing that dasatinib is active on a basal subtype of breast cancer cells [8,23]. Notably, gene expression profiling studies with a large set of breast tumors or cancer cell lines have identified basal epithelial types of tumors or cells that highly express *CK5*, *EGFR *and SFK *LYN *[24,25], which suggests that basal epithelial cells, as well as SFK and their cooperating partners, such as EGFR, have important roles in breast tumorigenesis [12].

In contrast to the well established and validated classification of breast cancer subtypes that has been used in the clinic to aid patient stratification [25,26], molecular classification of prostate cancer using genome-wide profiling techniques has been explored [27] but lacks validation with independent tumor cohorts. This may be a result of the heterogeneity of prostate tumors and the difficulty in obtaining biopsies from prostate cancer patients. It remains to be determined whether the molecular subtypes of prostate cancer identified through molecular profiling mimic the epithelial subtypes seen in normal prostatic epithelium, as is seen in breast cancer [25,26]. From our analysis of the data set published by Singh *et al*. [16], prostate cancer subtypes that express lower levels of *AR *and *PSA *and higher levels of *CK5 *(that is, the basal type) and those with the opposite expression pattern (that is, the luminal type) apparently exist. The derivation of prostate cancer cell lines such as MDAPCa2b, VCaP, DUCaP and LNCaP also demonstrates the existence of a luminal type of prostate cancer. The low expression levels of *PSA/AR *as well as *CK5 *in PC3 and DU145 cells may also suggest subtypes other than luminal and basal. It is noted that the subtype with high expression of *CK5 *and low expression of *AR *and *PSA *seen in our study is mainly based on immortalized prostatic cell lines. However, a recent study showed that sub-cell populations with varying degrees of differentiation co-exist in both *AR*-expressing LAPC-4/LAPC-9 human cancer xenograft models and PC3 and DU145 cancer cell populations. The sub-population that expressed CD44, another basal cell marker [28], showed the highest tumorigenicity [29]. These results strongly suggest that a subtype of cells with the phenotypes of normal basal cells exists in prostate tumors and may play a major role in determining tumor malignancy.

We found in our study that LNCaP cells, an androgen-sensitive prostate cancer cell line, exhibited sensitivity to dasatinib distinct from other androgen-sensitive cell lines, including 22Rv, MDAPCa2b, VCaP and DuCaP. Transcriptionally, these 5 cell lines resemble each other in terms of expression of *AR*/*PSA*, the 174 genes associated with *in vitro *response to dasatinib, as well as global gene expression (Figure 1c; other data not shown). It is not clear what mechanism in LNCaP cells caused dasatinib susceptibility. The *AR *gene mutation may be an appealing, but not necessarily straight-forward, explanation as LNCaP cells possess one T877A mutation and other cells either have no mutation (VCaP and DuCaP) or have other mutations (22Rv, H874Y) or additional types of mutations (MDAPCa2b, L701H and T877A) [30]. Alteration of the expression or sequence of *AR *may affect the function of AR in terms of binding to androgen [31] or cross-talk with growth factor and receptor pathways such as phosphorylation of AR by signaling cascades [32]. The inhibition of growth factor and receptor pathways by dasatinib may also induce the cells to re-establish the balance of signaling networks and modify the mode of action of AR. It is also possible that the function of one or more targets of dasatinib is indispensable for LNCaP cell growth.

By identifying genes whose expression was altered by drug treatment, we found a set of genes that may correlate with the mechanisms of action of dasatinib. This is desirable in drug development in two ways. First, markers correlated with mechanisms of action would enhance the level of confidence when biomarkers are used in clinical testing for the specific drug. Second, knowledge of drug efficacy through sensitive molecular testing can help to prevent premature discontinuation of clinical studies due to low clinical responses in early stage trials with small numbers of patients. In particular, while dasatinib is potent in inhibiting cell adhesion, migration and invasion, it appears in preclinical models to be cytostatic rather than cytotoxic. Thus, sensitive surrogate markers become critical for an evaluation of drug efficacy in early trials. In our study, we found that uPA, a downstream target of Src kinase, was modulated significantly by dasatinib, and such down-regulation was specifically caused by dasatinib and not by other cytotoxic agents, such as paclitaxel. In addition, the magnitude of drug-induced uPA reduction correlated very well with the sensitivity of cell lines to dasatinib, with higher reduction observed in sensitive cell lines and little or no change in resistant ones. These data suggest that uPA could potentially be used as a surrogate biomarker for monitoring the effect of dasatinib. More excitingly, uPA has been demonstrated to play an important role in prostatic tumorigenesis in numerous studies. For example, it is highly expressed in high grade prostate tumors and metastases [33], and when a tumor progresses to androgen independence, the level of uPA expression is enhanced [34]. In addition, RNA interference-induced knockdown of uPA inhibits invasion, survival and *in vivo *tumorigenicity of prostate cancer cells [35]. In the clinic, elevated uPA plasma level or uPA-uPA receptor densities are correlated with prostate cancer invasion, metastasis and poorer survival in prostate cancer [36,37] and its value as a prognostic marker has been well established in breast cancer [38,39].

Among the candidate predictive biomarker genes, several are components of signaling pathways important for cell survival and proliferation, such as the EGF-EGFR, transforming growth factor (TGF)-TGF receptor, fibroblast growth factor receptor and Met pathways. As an intracellular tyrosine kinase, Src can act as a signal transducer downstream of these receptor tyrosine kinases [11,12,40]. Alternatively, Src kinase may function independently of one or more pathways. Although the mechanisms of either co-operation or cross-talk of these pathways with the Src-mediated pathway in prostate cancer is not quite clear, they may still represent candidate target pathways for combination therapies to achieve additive or synergistic effects. This observation may provide insight for future clinical development strategies.

## Conclusion

Our study, utilizing a gene expression profiling approach in preclinical models, has identified prostatic biomarkers that are associated with sensitivity to dasatinib, a novel multi-targeted kinase inhibitor. In particular, five biomarkers (AR, PSA, CK5, uPA, and EphA2) could potentially help patient stratification and allow molecular monitoring of dasatinib activity in clinical trials. These markers are currently under early phase clinical evaluation using methods such as immunohistochemistry, ELISA or RT-PCR to identify potential associations with drug efficacy. In all, this preclinical study has provided a basis for clinical exploration, validation, and further implementation of a potential dasatinib efficacy signature for prostate cancer.

## Materials and methods

### Cell lines and cell culture

All cell lines were obtained from the American Type Culture Collection (Manassas, VA, USA), except DUCaP, which was obtained from Dr Kenneth Pienta at the University of Michigan. PWR1E and MDAPCa2b cells were grown in BRFF-HPC1 serum-free medium (AthenaES, Baltimore, MD, USA), and all other cells were cultured in RPMI 1640 supplemented with 10% fetal bovine serum, 100 IU/ml penicillin, and 100 mg/ml streptomycin (Invitrogen, Carlsbad, CA, USA). DUCaP cells were maintained in the presence of an immortalized mouse fibroblast cell line, which formed a layer beneath the DUCaP cells that easily dislodged. Cells were incubated at 37°C with circulation of 5% CO_2_.

### *In vitro *cell proliferation assay

The effect of dasatinib treatment on cell proliferation was measured using a tetrazolium compound-based colorimetric method (MTS kit, Promega, Madison, WI, USA). The optimum number of cells to seed in 96-well plates to achieve linearity was determined in pilot experiments. Cells were plated at a density of 2,000-5,000 cells/well into 96-well plates and cultured overnight. Cells were then treated with dasatinib at serially diluted concentrations. Three days later, the reagent solution was added to the medium and the absorbance was measured on a SpectraMax photometric plate reader (Molecular Devices, Sunnyvale, CA, USA) at 490 nm. The results were plotted against drug concentrations and IC_50 _values were calculated using Prism4 software (GraphPad, San Diego, CA, USA). The IC_50 _was the concentration of dasatinib that would reduce cell proliferation by 50% compared to control. A minimum of three independent assays were performed for each cell line. The mean ± standard deviation was calculated except for cell lines that were highly resistant to the compound, for which accurate IC_50 _values were hard to obtain. In the latter case, the concentration of dasatinib that was able to consistently reduce cell proliferation was used as the IC_50_. The inhibition of dasatinib on cell growth was also visually confirmed under the microscope. Dasatinib stock, dissolved in DMSO, was 10 mg/ml.

### Microarray analysis

Affymetrix HG-U133A 2.0 gene chips containing approximately 22,000 probe sets were used for gene expression profiling (Affymetrix, Santa Clara, CA, USA). Total RNA was isolated from the cells using the RNeasy kits (Qiagen, Valencia, CA, USA). The quality of the RNA was assessed using an Agilent 2100 Bioanalyzer (Agilent, Santa Clara, CA, USA). Total RNA (10 μg) was used for the preparation of biotin-labeled cRNA. Chip hybridization, scanning and data acquisition were performed according to the Expression Analysis Technical Manual provided by the manufacturer.

### Data analysis

The raw expression data were normalized using an RMA algorithm and analyzed in Partek Discovery Suite software (Partek, St Louis, MO, USA). Two statistical analyses, including one-way ANOVA (for comparison of gene expression between sensitive and resistant cell line groups) and Pearson correlation (between gene expression level and log_2_IC_50 _values) were performed to identify genes whose baseline expression levels correlated with sensitivity to dasatinib in 16 prostate cell lines (a *p *value < 0.05 in both analyses was required for inclusion). The gene list was further narrowed down by variation filters (10% CV of gene expression values across all samples and a minimum 3-fold differential expression between sensitive and resistant cell groups as defined by IC_50_). ESTs and gene duplicates were eliminated from the final list. Gene expression profiles of drug treated (100 nM for 2 days) cell lines were compared with those of DMSO control using paired *t*-test (*p *value < 0.05). Clustering analysis was performed using GeneCluster software and heatmaps were generated with red and green indicating high or low expression, respectively [41]. Gene expression raw data have been deposited in the Gene Expression Omnibus database (accession number GSE9633).

### Western blot analysis

Cell lysates were prepared from asynchronously growing cells using the RIPA buffer supplemented with protease (Roche Diagnostics, Indianapolis, IN, USA) and phosphatase inhibitor (Sigma, St Louis, MO, USA) cocktails. Protein concentration was determined using the BCA kit (Pierce, Rockford, IL, USA). Lystate (30 μg) was loaded and resolved on NuPAGE Novex 4-12% Bis-Tris gel (Invitrogen, Carlsbad, CA, USA). The blots were probed with mouse monoclonal anti-EphA2 (Upstate Biotechnology, Lake Placid, NY, USA) and anti-tubulin antibodies (Abcam, Cambridge, MA, USA) and developed with chemiluminescence reagent ECL Plus (GE Healthcare, Piscataway, NJ, USA).

### uPA protein ELISA assay

Cells were seeded in 24-well plates at a density of 25,000 cells per well. Two days later, the cells were washed twice with phosphate-buffered saline and the medium was changed to RPMI 1640 containing 0.1% fetal bovine serum and different concentrations of dasatinib or paclitaxel. Medium was sampled at 0, 2, 4, 8, 24, and 48 h and immediately centrifuged at 10,000 g for 5 minutes. The supernatants were frozen at -80°C until analysis. The total number of cells was quantified using a cell counter, and the number of viable cells was assessed with Trypan Blue. The amount of uPA protein in the supernatant was determined using the uPA ELISA kit (America Diagnostica, Stamford, CT, USA), and the concentrations of uPA secreted by 50,000 viable cells into the medium were calculated.

## Abbreviations

AR, androgen receptor; CK, cytokeratin; CV, coefficient of variation; EGFR, epidermal growth factor receptor; ELISA, enzyme-linked immunosorbent assay; EST, expressed sequence tag; IC_50_, half maximal inhibitory concentration; PDGFR, platelet-derived growth factor receptor; PSA, prostate-specific antigen (also known as kallikrein 3); SFK, Src-family kinase; TGF, transforming growth factor; uPA, urokinase-type plasminogen activator.

## Authors' contributions

XDW designed the study, performed data analyses, and wrote the manuscript. KR performed the EphA2 Western blot analysis. FRL contributed data on the down-regulation of uPA protein in PC3 cells following drug treatment. LAX provided statistical assistance. FL shared insight and provided support for the study. EC helped conceive the study and edited the manuscript. FH helped conceive the study, advised on study design, and edited the manuscript.

## Additional data files

The following additional data are available with the online version of this paper. Additional data file 1 is a table listing biomarkers correlated with sensitivity or resistance to dasatinib. Additional data file 2 is a table listing common predictive markers identified in prostate and breast preclinical models. Additional data file 3 provides microarray data on baseline gene expression of cell lines used for identification of genes whose expression correlated with *in vitro *sensitivity to dasatinib. Additional data file 4 provides microarray data on gene expression of cell lines treated with dasatinib or DMSO control.

## Supplementary Material

Additional data file 1 Biomarkers correlated with sensitivity or resistance to dasatinib. Click here for file

Additional data file 2 Common predictive markers identified in prostate and breast preclinical models. Click here for file

Additional data file 3 The hybridization data (in log scale) were normalized with the RMA algorithm. The IC_50 _in nM and log_2_IC50 values as well as the classification of cell lines as resistant (R) or sensitive (S) are also provided at the top of the spreadsheet. Click here for file

Additional data file 4 These data were used for identification of genes whose expression was modulated by dasatinib. The hybridization data (in log scale) were normalized with the RMA algorithm. The treatment and the classification of cell lines as resistant (R) or sensitive (S) are also provided at the top of the spreadsheet. Click here for file
