# Supplementary material for: Identification of candidate predictive and surrogate molecular markers for dasatinib in prostate cancer: rationale for patient selection and efficacy monitoring
Source: Genome Biol. 2007 Nov 29;8(11):R255. doi: 10.1186/gb-2007-8-11-r255 (PMC2258199; doi:10.1186/gb-2007-8-11-r255)
Supplement: Additional data file 1 — Biomarkers correlated with sensitivity or resistance to dasatinib. [file gb-2007-8-11-r255-S1.doc]

Table 1. Biomarkers correlated with sensitivity or resistance to dasatinib

| Probe ID | Gene description | Gene Symbol | p-value (1-way ANOVA) | p-value (IC50 correlation) | Fold Change | Dasatinib Modulation  (p<0.05) |
| --- | --- | --- | --- | --- | --- | --- |
| **Genes expressed higher in sensitive cells:** | | | | | | |
| 204855_at | serpin peptidase inhibitor, clade B, member 5 | SERPINB5 | 1.75E-05 | 5.24E-04 | 6.91 |  |
| 209125_at | keratin 6 | KRT6 | 3.47E-03 | 3.01E-02 | 6.46 |  |
| 206884_s_at | sciellin | SCEL | 4.11E-04 | 4.33E-03 | 5.8 | decreased |
| 204748_at | prostaglandin-endoperoxide synthase 2 | PTGS2 | 1.10E-02 | 4.22E-02 | 5.3 |  |
| 204268_at | S100 calcium binding protein A2 | S100A2 | 3.15E-04 | 4.90E-03 | 5.2 |  |
| 209016_s_at | keratin 7 | KRT7 | 8.48E-05 | 1.38E-03 | 5.02 |  |
| 206295_at | interleukin 18 | IL18 | 2.37E-04 | 4.76E-03 | 4.6 |  |
| 209369_at | annexin A3 | ANXA3 | 4.59E-04 | 4.18E-03 | 4.46 | decreased |
| 41469_at | peptidase inhibitor 3, skin-derived | PI3 | 1.69E-02 | 2.74E-02 | 4.44 |  |
| 219936_s_at | G protein-coupled receptor 87 | GPR87 | 7.86E-04 | 9.78E-03 | 4.42 |  |
| 201820_at | keratin 5 | CK5 | 5.67E-03 | 4.91E-02 | 4.41 |  |
| 205239_at | amphiregulin | AREG | 5.32E-05 | 4.62E-04 | 4.38 |  |
| 204614_at | serpin peptidase inhibitor, clade B, member 2 | SERPINB2 | 2.44E-02 | 4.85E-02 | 4.36 |  |
| 203887_s_at | thrombomodulin | THBD | 5.59E-03 | 3.21E-02 | 4.23 |  |
| 206595_at | cystatin E/M | CST6 | 5.86E-04 | 8.19E-03 | 4.21 | decreased |
| 204455_at | dystonin | DST | 4.25E-03 | 3.09E-02 | 4.19 |  |
| 203323_at | caveolin 2 | CAV2 | 1.12E-03 | 4.32E-03 | 4.09 |  |
| 212531_at | lipocalin 2 | LCN2 | 3.21E-03 | 7.33E-03 | 4.03 |  |
| 219529_at | chloride intracellular channel 3 | CLIC3 | 1.45E-03 | 1.15E-02 | 3.96 |  |
| 209909_s_at | transforming growth factor, beta 2 | TGFB2 | 6.38E-04 | 8.25E-03 | 3.94 |  |
| 203074_at | annexin A8 | ANXA8 | 3.50E-03 | 4.12E-02 | 3.93 |  |
| 203153_at | interferon-induced protein with tetratricopeptide repeats 1 | IFIT1 | 1.33E-03 | 2.56E-03 | 3.92 |  |
| 211756_at | parathyroid hormone-like hormone | PTHLH | 2.60E-02 | 2.88E-02 | 3.91 |  |
| 203066_at | B cell RAG associated protein | GALNAC4S-6ST | 5.57E-05 | 3.89E-04 | 3.91 |  |
| 218182_s_at | claudin 1 | CLDN1 | 1.54E-03 | 1.86E-02 | 3.91 |  |
| 202411_at | interferon, alpha-inducible protein 27 | IFI27 | 5.44E-03 | 1.76E-02 | 3.89 |  |
| 205014_at | fibroblast growth factor binding protein 1 | FGFBP1 | 5.26E-03 | 1.58E-02 | 3.86 |  |
| 203535_at | S100 calcium binding protein A9 | S100A9 | 2.55E-02 | 4.12E-02 | 3.83 |  |
| 204439_at | interferon-induced protein 44-like | IFI44L | 1.33E-02 | 2.40E-02 | 3.82 |  |
| 206504_at | cytochrome P450, family 24, subfamily A, polypeptide 1 | CYP24A1 | 1.01E-02 | 2.52E-02 | 3.82 |  |
| 201842_s_at | EGF-containing fibulin-like extracellular matrix protein 1 | EFEMP1 | 5.26E-03 | 2.92E-02 | 3.81 |  |
| 214022_s_at | interferon induced transmembrane protein 1 | IFITM1 | 4.05E-04 | 4.14E-03 | 3.73 |  |
| 202267_at | laminin, gamma 2 | LAMC2 | 9.70E-04 | 8.46E-03 | 3.71 | decreased |
| 219836_at | zinc finger, BED-type containing 2 | ZBED2 | 2.04E-03 | 2.13E-02 | 3.7 | decreased |
| 205627_at | cytidine deaminase | CDA | 2.82E-04 | 1.75E-03 | 3.69 |  |
| 209301_at | carbonic anhydrase II | CA2 | 4.60E-03 | 2.56E-02 | 3.68 |  |
| 214453_s_at | interferon-induced protein 44 | IFI44 | 1.14E-03 | 3.42E-03 | 3.67 |  |
| 206332_s_at | interferon, gamma-inducible protein 16 | IFI16 | 1.19E-02 | 3.35E-02 | 3.63 |  |
| 202086_at | myxovirus resistance 1 | MX1 | 2.04E-03 | 4.92E-03 | 3.62 |  |
| 213139_at | snail homolog 2 | SNAI2 | 9.26E-04 | 6.16E-03 | 3.62 |  |
| 203065_s_at | caveolin 1 | CAV1 | 2.29E-03 | 8.77E-03 | 3.61 |  |
| 209774_x_at | chemokine (C-X-C motif) ligand 2 | CXCL2 | 1.52E-02 | 2.03E-02 | 3.55 |  |
| 205767_at | epiregulin | EREG | 3.84E-03 | 1.01E-02 | 3.54 | decreased |
| 212657_s_at | interleukin 1 receptor antagonist | IL1RN | 1.22E-02 | 3.01E-02 | 3.54 |  |
| 219691_at | sterile alpha motif domain containing 9 | SAMD9 | 9.35E-03 | 4.13E-02 | 3.51 |  |
| 200600_at | moesin | MSN | 4.02E-04 | 1.04E-03 | 3.48 |  |
| 209949_at | neutrophil cytosolic factor 2 | NCF2 | 5.09E-03 | 1.94E-02 | 3.45 |  |
| 205569_at | lysosomal-associated membrane protein 3 | LAMP3 | 3.17E-03 | 6.38E-03 | 3.44 |  |
| 208949_s_at | lectin, galactoside-binding, soluble, 3 | LGALS3 | 4.65E-03 | 1.85E-02 | 3.4 |  |
| 218677_at | S100 calcium binding protein A14 | S100A14 | 2.04E-03 | 1.72E-02 | 3.39 |  |
| 206421_s_at | serpin peptidase inhibitor, clade B, member 7 | SERPINB7 | 8.26E-03 | 1.69E-02 | 3.38 |  |
| 202686_s_at | AXL receptor tyrosine kinase | AXL | 7.82E-03 | 2.10E-02 | 3.36 | decreased |
| 222242_s_at | kallikrein 5 | KLK5 | 7.36E-03 | 2.71E-02 | 3.35 |  |
| 202949_s_at | four and a half LIM domains 2 | FHL2 | 6.41E-03 | 3.23E-02 | 3.31 | decreased |
| 210933_s_at | fascin homolog 1 | FSCN1 | 1.28E-03 | 5.84E-03 | 3.31 |  |
| 214639_s_at | homeo box A1 | HOXA1 | 4.05E-04 | 3.61E-03 | 3.31 |  |
| 202869_at | 2',5'-oligoadenylate synthetase 1 | OAS1 | 9.56E-03 | 1.77E-02 | 3.31 |  |
| 204602_at | dickkopf homolog 1 | DKK1 | 8.60E-03 | 2.22E-02 | 3.31 |  |
| 209278_s_at | tissue factor pathway inhibitor 2 | TFPI2 | 1.77E-02 | 2.41E-02 | 3.29 |  |
| 210511_s_at | inhibin, beta A | INHBA | 1.04E-02 | 3.94E-02 | 3.29 |  |
| 204400_at | embryonal Fyn-associated substrate | EFS | 1.47E-02 | 3.00E-02 | 3.28 |  |
| 201631_s_at | immediate early response 3 | IER3 | 2.45E-03 | 9.30E-03 | 3.28 |  |
| 205479_s_at | plasminogen activator, urokinase | UPA | 5.66E-04 | 8.12E-04 | 3.27 | decreased |
| 213816_s_at | met proto-oncogene | MET | 1.54E-03 | 8.71E-03 | 3.26 |  |
| 209270_at | laminin, beta 3 | LAMB3 | 4.23E-05 | 5.06E-04 | 3.26 |  |
| 217767_at | complement component 3 | C3 | 1.94E-03 | 1.93E-02 | 3.25 |  |
| 201641_at | bone marrow stromal cell antigen 2 | BST2 | 9.99E-03 | 4.78E-02 | 3.25 |  |
| 205016_at | transforming growth factor, alpha | TGFA | 5.51E-03 | 2.83E-02 | 3.25 |  |
| 209651_at | transforming growth factor beta 1 induced transcript 1 | TGFB1I1 | 1.01E-02 | 4.60E-02 | 3.24 |  |
| 202565_s_at | supervillin | SVIL | 5.86E-04 | 7.34E-03 | 3.23 |  |
| 211864_s_at | fer-1-like 3, myoferlin | FER1L3 | 5.16E-03 | 1.65E-02 | 3.23 |  |
| 206561_s_at | aldo-keto reductase family 1, member B10 | AKR1B10 | 1.65E-02 | 1.56E-02 | 3.21 |  |
| 219412_at | RAB38, member RAS oncogene family | RAB38 | 4.70E-03 | 2.54E-02 | 3.2 |  |
| 205349_at | guanine nucleotide binding protein | GNA15 | 2.34E-03 | 1.96E-02 | 3.2 |  |
| 208944_at | transforming growth factor, beta receptor II | TGFBR2 | 2.13E-03 | 1.35E-02 | 3.19 |  |
| 214620_x_at | peptidylglycine alpha-amidating monooxygenase | PAM | 3.97E-03 | 1.76E-02 | 3.17 |  |
| 210074_at | cathepsin L2 | CTSL2 | 3.17E-05 | 1.39E-03 | 3.17 |  |
| 209493_at | PDZ domain containing 3 | PDZK3 | 3.19E-03 | 1.67E-02 | 3.16 |  |
| 204989_s_at | integrin, beta 4 | ITGB4 | 1.76E-04 | 3.28E-03 | 3.16 |  |
| 217996_at | pleckstrin homology-like domain, family A, member 1 | PHLDA1 | 9.17E-03 | 1.62E-02 | 3.15 |  |
| 204415_at | interferon, alpha-inducible protein | G1P3 | 5.98E-04 | 1.95E-03 | 3.13 |  |
| 203638_s_at | fibroblast growth factor receptor 2 | FGFR2 | 7.62E-03 | 4.20E-02 | 3.13 |  |
| 203407_at | periplakin | PPL | 2.49E-04 | 4.47E-03 | 3.12 |  |
| 219403_s_at | heparanase | HPSE | 5.48E-04 | 3.67E-03 | 3.12 |  |
| 203964_at | N-myc (and STAT) interactor | NMI | 3.12E-03 | 1.20E-02 | 3.12 |  |
| 202430_s_at | phospholipid scramblase 1 | PLSCR1 | 8.22E-03 | 2.41E-02 | 3.1 |  |
| 201858_s_at | proteoglycan 1, secretory granule | PRG1 | 4.77E-02 | 4.19E-02 | 3.1 |  |
| 203476_at | trophoblast glycoprotein | TPBG | 9.40E-03 | 2.96E-02 | 3.09 |  |
| 218943_s_at | DEAD (Asp-Glu-Ala-Asp) box polypeptide 58 | DDX58 | 1.32E-03 | 5.71E-03 | 3.09 |  |
| 203939_at | 5'-nucleotidase, ecto (CD73) | NT5E | 3.82E-03 | 7.69E-03 | 3.09 |  |
| 203372_s_at | suppressor of cytokine signaling 2 | SOCS2 | 7.70E-05 | 1.00E-04 | 3.08 |  |
| 210138_at | regulator of G-protein signalling 20 | RGS20 | 1.81E-03 | 1.20E-02 | 3.08 |  |
| 201487_at | cathepsin C | CTSC | 6.81E-03 | 2.15E-02 | 3.07 |  |
| 213906_at | v-myb myeloblastosis viral oncogene homolog (avian)-like 1 | MYBL1 | 7.99E-03 | 1.89E-02 | 3.07 |  |
| 39402_at | interleukin 1, beta | IL1B | 2.77E-02 | 3.45E-02 | 3.07 |  |
| 203851_at | insulin-like growth factor binding protein 6 | IGFBP6 | 1.34E-02 | 2.21E-02 | 3.07 |  |
| 206156_at | gap junction protein, beta 5 (connexin 31.1) | GJB5 | 3.68E-03 | 3.61E-02 | 3.07 |  |
| 213711_at | keratin, hair, basic, 1 | KRTHB1 | 6.73E-03 | 1.08E-02 | 3.07 |  |
| 219901_at | FYVE, RhoGEF and PH domain containing 6 | FGD6 | 3.06E-04 | 1.83E-03 | 3.06 |  |
| 220658_s_at | aryl hydrocarbon receptor nuclear translocator-like 2 | ARNTL2 | 4.15E-03 | 4.71E-03 | 3.06 | decreased |
| 204972_at | 2'-5'-oligoadenylate synthetase 2, 69/71kDa | OAS2 | 3.75E-02 | 3.95E-02 | 3.06 |  |
| 201312_s_at | SH3 domain binding glutamic acid-rich protein like | SH3BGRL | 8.44E-03 | 7.12E-03 | 3.06 |  |
| 219032_x_at | opsin 3 (encephalopsin, panopsin) | OPN3 | 4.03E-03 | 5.85E-03 | 3.06 |  |
| 202546_at | vesicle-associated membrane protein 8 | VAMP8 | 1.09E-03 | 2.74E-03 | 3.06 |  |
| 201012_at | annexin A1 | ANXA1 | 7.84E-03 | 2.98E-02 | 3.05 |  |
| 203695_s_at | deafness, autosomal dominant 5 | DFNA5 | 7.57E-04 | 4.90E-03 | 3.05 |  |
| 203021_at | secretory leukocyte peptidase inhibitor | SLPI | 4.01E-03 | 1.32E-02 | 3.05 |  |
| 202833_s_at | serpin peptidase inhibitor, clade A, member 1 | SERPINA1 | 4.93E-02 | 3.16E-02 | 3.04 |  |
| 203585_at | zinc finger protein 185 (LIM domain) | ZNF185 | 2.33E-04 | 8.85E-04 | 3.04 |  |
| 212724_at | Rho family GTPase 3 | RND3 | 1.07E-02 | 3.81E-02 | 3.04 |  |
| 203820_s_at | IGF-II mRNA-binding protein 3 | IMP-3 | 7.38E-03 | 2.08E-02 | 3.04 |  |
| 201325_s_at | epithelial membrane protein 1 | EMP1 | 1.14E-02 | 3.97E-02 | 3.03 |  |
| 202286_s_at | tumor-associated calcium signal transducer 2 | TACSTD2 | 4.98E-03 | 2.63E-02 | 3.02 |  |
| 209765_at | ADAM metallopeptidase domain 19 | ADAM19 | 9.42E-03 | 3.24E-02 | 3.02 |  |
| 200923_at | lectin, galactoside-binding, soluble, 3 binding protein | LGALS3BP | 5.54E-04 | 5.87E-03 | 3.02 |  |
| 203256_at | cadherin 3, type 1, P-cadherin | CDH3 | 1.46E-03 | 1.05E-02 | 3.02 |  |
| 204734_at | keratin 15 | KRT15 | 4.20E-03 | 2.37E-02 | 3.02 |  |
| 215465_at | ATP-binding cassette, sub-family A (ABC1), member 12 | ABCA12 | 1.52E-02 | 2.79E-02 | 3 |  |
| 202644_s_at | tumor necrosis factor, alpha-induced protein 3 | TNFAIP3 | 2.71E-03 | 1.35E-02 | 3 |  |
| 209946_at | vascular endothelial growth factor C | VEGFC | 6.89E-03 | 1.48E-02 | 3 |  |
| 203882_at | interferon-stimulated transcription factor 3, gamma | ISGF3G | 3.43E-04 | 2.19E-03 | 3 |  |
| 218854_at | squamous cell carcinoma antigen recognized by T cells 2 | SART2 | 6.21E-03 | 3.33E-02 | 2.99 |  |
| 212192_at | potassium channel tetramerisation domain containing 12 | KCTD12 | 1.69E-02 | 4.51E-02 | 2.99 |  |
| 204470_at | chemokine (C-X-C motif) ligand 1 | CXCL1 | 3.53E-02 | 3.92E-02 | 2.99 |  |
| **Genes expressed higher in resistant cells:** | | | | | | |
| 203824_at | tetraspanin 8 | TSPAN8 | 2.73E-05 | 5.27E-04 | 5.41 |  |
| 214774_x_at | trinucleotide repeat containing 9 | TNRC9 | 7.44E-05 | 4.01E-04 | 5.1 |  |
| 209854_s_at | kallikrein 2, prostatic | KLK2 | 1.86E-03 | 5.88E-03 | 4.84 |  |
| 211144_x_at | T cell receptor gamma constant 2 | TRGC2 | 1.91E-03 | 6.58E-03 | 4.6 |  |
| 211298_s_at | albumin | ALB | 3.38E-03 | 8.64E-03 | 4.52 |  |
| 207802_at | cysteine-rich secretory protein 3 | CRISP3 | 1.58E-02 | 2.15E-02 | 4.35 |  |
| 206167_s_at | Rho GTPase activating protein 6 | ARHGAP6 | 9.90E-04 | 6.09E-03 | 4.25 |  |
| 221558_s_at | lymphoid enhancer-binding factor 1 | LEF1 | 4.71E-06 | 3.74E-05 | 3.93 |  |
| 221019_s_at | collectin sub-family member 12 | COLEC12 | 1.06E-05 | 1.61E-04 | 3.88 |  |
| 215363_x_at | folate hydrolase (prostate-specific membrane antigen) 1 | FOLH1 | 1.29E-02 | 3.81E-02 | 3.76 |  |
| 206001_at | neuropeptide Y | NPY | 1.93E-02 | 2.60E-02 | 3.74 |  |
| 210576_at | cytochrome P450, family 4, subfamily F, polypeptide 8 | CYP4F8 | 2.80E-03 | 3.59E-03 | 3.7 |  |
| 211621_at | androgen receptor | AR | 1.31E-03 | 4.66E-03 | 3.68 |  |
| 205110_s_at | fibroblast growth factor 13 | FGF13 | 3.08E-03 | 1.34E-02 | 3.61 |  |
| 210755_at | hepatocyte growth factor | HGF | 2.24E-04 | 6.10E-04 | 3.57 |  |
| 206214_at | phospholipase A2, group VII | PLA2G7 | 6.02E-04 | 5.21E-03 | 3.56 |  |
| 204583_x_at | kallikrein 3 (prostate specific antigen) | KLK3 | 1.33E-02 | 2.70E-02 | 3.51 |  |
| 203680_at | protein kinase, cAMP-dependent, regulatory, type II, beta | PRKAR2B | 3.56E-04 | 3.75E-03 | 3.46 |  |
| 204393_s_at | acid phosphatase, prostate | ACPP | 6.94E-03 | 1.68E-02 | 3.45 |  |
| 219197_s_at | signal peptide, CUB domain, EGF-like 2 | SCUBE2 | 1.86E-03 | 4.07E-03 | 3.43 |  |
| 219584_at | phospholipase A1 member A | PLA1A | 2.71E-02 | 4.47E-02 | 3.42 |  |
| 202747_s_at | integral membrane protein 2A | ITM2A | 1.88E-02 | 2.70E-02 | 3.42 |  |
| 219196_at | secretogranin III | SCG3 | 2.24E-03 | 4.82E-03 | 3.39 |  |
| 209785_s_at | phospholipase A2, group IVC | PLA2G4C | 3.30E-03 | 5.78E-03 | 3.37 |  |
| 213541_s_at | v-ets erythroblastosis virus E26 oncogene like | ERG | 2.09E-02 | 3.31E-02 | 3.37 |  |
| 221018_s_at | tudor domain containing 1 | TDRD1 | 2.31E-03 | 4.25E-03 | 3.36 |  |
| 205942_s_at | acyl-CoA synthetase medium-chain family member 3 | ACSM3 | 3.62E-03 | 1.99E-02 | 3.35 |  |
| 212224_at | aldehyde dehydrogenase 1 family, member A1 | ALDH1A1 | 8.50E-03 | 1.94E-02 | 3.26 |  |
| 205938_at | protein phosphatase 1E | PPM1E | 2.86E-04 | 2.14E-03 | 3.24 |  |
| 221523_s_at | Ras-related GTP binding D | RRAGD | 1.43E-03 | 6.19E-03 | 3.24 |  |
| 204337_at | regulator of G-protein signalling 4 | RGS4 | 1.01E-02 | 2.63E-02 | 3.23 |  |
| 33767_at | neurofilament, heavy polypeptide | NEFH | 7.68E-04 | 1.32E-03 | 3.22 |  |
| 206664_at | sucrase-isomaltase | SI | 1.58E-02 | 2.51E-02 | 3.21 |  |
| 206505_at | UDP glucuronosyltransferase 2 family, polypeptide B4 | UGT2B4 | 5.51E-03 | 1.02E-02 | 3.18 |  |
| 214156_at | myosin VIIA and Rab interacting protein | MYRIP | 7.82E-03 | 2.40E-02 | 3.17 |  |
| 206349_at | leucine-rich, glioma inactivated 1 | LGI1 | 2.42E-02 | 3.40E-02 | 3.15 |  |
| 222317_at | Phosphodiesterase 3B, cGMP-inhibited | PDE3B | 2.79E-03 | 1.34E-02 | 3.15 |  |
| 213712_at | elongation of very long chain fatty acids-like 2 | ELOVL2 | 3.11E-04 | 2.76E-03 | 3.13 |  |
| 205850_s_at | gamma-aminobutyric acid (GABA) A receptor, beta 3 | GABRB3 | 2.80E-03 | 1.24E-02 | 3.12 |  |
| 209869_at | adrenergic, alpha-2A-, receptor | ADRA2A | 9.22E-03 | 2.54E-02 | 3.11 |  |
| 219312_s_at | zinc finger and BTB domain containing 10 | ZBTB10 | 5.94E-03 | 4.05E-02 | 3.1 |  |
| 214087_s_at | myosin binding protein C, slow type | MYBPC1 | 6.78E-04 | 2.10E-03 | 3.09 |  |
| 205357_s_at | angiotensin II receptor, type 1 | AGTR1 | 3.01E-03 | 5.83E-03 | 3.08 |  |
| 205221_at | homogentisate 1,2-dioxygenase | HGD | 1.55E-03 | 4.28E-03 | 3.08 |  |
| 203485_at | reticulon 1 | RTN1 | 2.51E-02 | 4.68E-02 | 3.07 |  |
| 203130_s_at | kinesin family member 5C | KIF5C | 5.35E-03 | 9.48E-03 | 3.06 |  |
| 214913_at | ADAM metallopeptidase with thrombospondin type 1 motif, 3 | ADAMTS3 | 1.31E-03 | 5.82E-03 | 3.03 |  |
| 204714_s_at | coagulation factor V (proaccelerin, labile factor) | F5 | 1.46E-02 | 3.10E-02 | 3.02 |  |
| 219915_s_at | solute carrier family 16 (monocarboxylic acid transporters), member 10 | SLC16A10 | 1.63E-03 | 4.08E-03 | 3.01 |  |
| 205979_at | secretoglobin, family 2A, member 1 | SCGB2A1 | 2.84E-03 | 5.38E-03 | 2.99 |  |
