# Supplementary material for: Identification of candidate predictive and surrogate molecular markers for dasatinib in prostate cancer: rationale for patient selection and efficacy monitoring
Source: Genome Biol. 2007 Nov 29;8(11):R255. doi: 10.1186/gb-2007-8-11-r255 (PMC2258199; doi:10.1186/gb-2007-8-11-r255)
Supplement: Additional data file 2 — Common predictive markers identified in prostate and breast preclinical models. [file gb-2007-8-11-r255-S2.doc]

Table 2. Common predictive markers identified in prostate and breast preclinical models

| Probe ID | Gene symbol | Gene description | p-value  (1-way ANOVA) | p-value  (IC50 correlation) | Fold change |
| --- | --- | --- | --- | --- | --- |
| 203324_s_at | CAV2 | caveolin 2 | 1.21E-03 | 5.74E-03 | 4.63 |
| 212097_at | CAV1 | caveolin 1 | 2.74E-03 | 1.00E-02 | 4.19 |
| 211668_s_at* | UPA | plasminogen activator, urokinase | 1.02E-03 | 1.50E-03 | 3.80 |
| 213139_at | SNAI2 | snail homolog 2 | 9.26E-04 | 6.16E-03 | 3.62 |
| 200600_at | MSN | moesin | 4.02E-04 | 1.04E-03 | 3.48 |
| 203510_at | MET | met proto-oncogene | 4.93E-03 | 1.97E-02 | 3.30 |
| 208966_x_at | IFI16 | interferon, gamma-inducible protein 16 | 1.64E-02 | 4.34E-02 | 3.27 |
| 209270_at | LAMB3 | laminin, beta 3 | 4.23E-05 | 5.06E-04 | 3.26 |
| 208944_at | TGFBR2 | transforming growth factor, beta receptor II | 2.13E-03 | 1.35E-02 | 3.19 |
| 201012_at | ANXA1 | annexin A1 | 7.84E-03 | 2.98E-02 | 3.05 |
| 212192_at | KCTD12 | potassium channel tetramerisation domain containing 12 | 1.69E-02 | 4.51E-02 | 2.99 |
| 203499_at | EPHA2 | EPH receptor A2 | 5.35E-03 | 2.02E-02 | 2.69 |
| 219926_at | POPDC3 | popeye domain containing 3 | 3.25E-02 | 2.72E-02 | 2.53 |
| 212510_at | GPD1L | glycerol-3-phosphate dehydrogenase 1-like | 1.21E-02 | 4.41E-02 | 0.42** |

* in addition to the first probe set shown in Table 1, data of a second probe set for UPA are shown.

** indicates lower expression in sensitive than in resistant cell lines.
